# Refractive Errors, Amplitude of Accommodation, and Myopia Progression in Kazakhstani Medical Students: 5-Year Follow-Up

**DOI:** 10.3390/jcm13133985

**Published:** 2024-07-08

**Authors:** Yuliya Semenova, Malika Urazhanova, Lisa Lim, Nazerke Kaiyrzhanova

**Affiliations:** 1 Department of Surgery, Nazarbayev University School of Medicine, Astana 010000, Kazakhstan; 2 Department of Neurology, Ophthalmology, and Otorhinolaryngology, Semey Medical University, Semey 071400, Kazakhstan; malika071088@gmail.com; 3 Nazarbayev University Graduate School of Public Policy, Astana 010000, Kazakhstan; lisa.lim@nu.edu.kz; 4 SemeyOFTUM Ophthalmology Clinic, Semey 071400, Kazakhstan; naz_ssma@mail.ru

**Keywords:** refractive errors, myopia, amplitude of accommodation, medical students

## Abstract

**Background/Objectives:** this longitudinal study aimed to investigate the refractive errors, the amplitude of accommodation, and myopia progression in Kazakhstani medical students as they progressed from the first to the fifth course of their studies. **Methods:** A total of 696 students from Semey Medical University underwent non-cycloplegic and cycloplegic autorefraction in the first course, and 655 were available for examination in the fifth year of study. The amplitude of accommodation was measured before the instillation of cycloplegics using the push-up and push-down methods. A self-administered questionnaire was applied to evaluate the risk factors associated with myopia progression. **Results:** In the first course, the median spherical equivalent was −0.75 Diopters before cycloplegia and −0.25 Diopters after cycloplegia. In the fifth course, it constituted −1.125 Diopters before cycloplegia and −0.5 Diopters after cycloplegia. The proportion of students with myopia following cycloplegic refraction increased from 44.7% in the first course to 47.5% in the fifth course. The proportion of emmetropic students declined from 31.5% to 30.3%, and hyperopia decreased from 23.8% to 16.8%. The dioptric power of accommodative excess increased from 0.375 in the first year to 0.50 in the fifth year. The hours spent on near-work activities, such as reading books, writing, working at a computer, and using a mobile device, were significantly associated with a myopia progression of ≥0.5 Diopters. **Conclusions:** the findings of this study suggest implications for public health policy and educational practice.

## 1. Introduction

Refractive errors, particularly myopia, are the leading causes of low vision worldwide. The prevalence of myopia is on the rise, with projections suggesting that by 2050, nearly 50% of the world population will be affected, including up to 10% with high myopia [1]. Myopia not only exhibits an upward trend in rates but also affects individuals at a younger age. The earlier myopia initiates, the greater the resulting degree of refractive error, underscoring the importance of addressing this issue promptly [2]. Myopia not only diminishes the quality of life and raises the risk of disabling ocular disorders but also creates an economic burden due to the necessity of frequent and long-term management [3].

Typically, myopia begins its development in childhood, with onset commonly occurring between 6 and 14 years. Progression continues during childhood and young adolescence, with stabilization expected around 15–16 years of age [4]. Rates of myopia vary across different nations, with the highest rates observed in East and Southeast Asia, attributed to genetic factors and high educational overload. In these parts of the world, myopia often starts already at 5–6 years of age and progresses to −5 and −6 Diopters by the age of 11–13 years [5]. Myopia is less common in Central Europe, Central Asia, and Central Africa, where it was reported to affect 27.1%, 17.0%, and 7.0% of the population, respectively [1].

There is an abundance of academic research investigating the incidence rates of myopia among school-age children, with several meta-analyses published on the topic [6,7,8]. However, less is known about incidences in adults and, particularly, young adults. This is due to the common perception of young adulthood being a period when vision and eye health are at their peak, myopia has stabilized, and the rate of disabling ocular disorders is low [9]. Consequently, compared to children, the epidemiology of refractive errors in young adults is insufficiently studied, highlighting the need to address this research gap.

The period of university studies represents another phase of life characterized by a demanding visual workload, potentially contributing to the onset of adult myopia or the progression of pre-existing myopia. Students pursuing academic degrees often encounter visually intensive tasks integral to the curriculum. While several longitudinal studies have explored myopia progression rates in university students with varying durations of follow-up [10,11,12,13], there remains a gap in research specifically addressing medical students. 

This student population deserves particular attention for several reasons. First, medical students face a highly demanding visual workload due to the rigorous nature of their studies. Their curriculum typically involves extensive reading, computer use, and other visually intensive tasks. In addition, medical education commonly spans many years, subjecting students to prolonged exposure to high visual demands, which can induce myopia or exacerbate its progression over time. It is, therefore, critical to study the ocular problems encountered by medical students throughout their education [10]. Given the limited knowledge regarding the rate of myopia progression during the entire duration of medical school studies, this study aims to fill the existing gap. Therefore, this longitudinal study aimed to investigate the refractive errors, amplitude of accommodation, and myopia progression in Kazakhstani medical students as they advance from the first to the fifth course of their studies.

## 2. Materials and Methods

### 2.1. Study Design and Procedures

This study was conducted at Semey Medical University (SMU), one of the largest medical schools in Kazakhstan, located in Semey City, Abay region. All domestic and international first-year students from the departments of Medicine, Public Health, Dentistry, Nursing, and Pharmacy were invited to participate in free refraction testing during the first two months (September and October) of their study at the University. Inclusion criteria considered for participation were providing informed consent, having no past history of allergy to mydriatics, and the absence of ocular disorders, trauma, and surgery. Of the 750 students, 696 agreed to participate, resulting in a response rate of 92.8%. Six hundred and fifty-five (655) students were available for examination in the fifth year of study. The same inclusion/exclusion criteria were applied at the 5-year follow-up. Apart from refraction testing, students were administered a self-reported questionnaire to elucidate the risk factors associated with myopia progression.

The study received approval from the Ethics Committee of Semey Medical University (Protocol #35, dated 15 December 2015).

### 2.2. Refraction Measurement

The same approach for refraction measurement was followed at baseline and at the 5-year follow-up. Objective refraction was evaluated twice, before cycloplegia and after. Cycloplegia was achieved by the instillation of 1% tropicamide eye drops (Rompharm, Otopeni, Romania) into both eyes, followed by the instillation of 1% cyclopentolate (Promed Exports, Green Park, Delhi, India) at a 5 min interval. Refraction was measured 30 min after the instillation of the second eye drop using the Medizs RK-11 autorefractometer (Huvitz, Yongin, Republic of Korea). Three measurements of refraction were performed, and the average of the three readings was recorded.

The spherical equivalent (SE) was calculated as the sum of the spherical component and half of the cylindrical component [14]. Eyes with SE from −0.49 to +0.49 Diopters were categorized as emmetropic, eyes with SE of −0.5 Diopters and greater were classified as myopic, while eyes with SE of +0.5 Diopters and greater were categorized as hyperopic. Low myopia was defined as SE ranging between −0.5 and −2.99 Diopters, moderate myopia was defined as SE ranging between −3.0 and −5.99 Diopters, and high myopia was defined as SE of −6.0 Diopters and greater. Similarly, hyperopia was considered to be low if SE was between +0.5 and +2.99 Diopters, moderate hyperopia was defined as SE ranging between +3.0 and +5.99 Diopters, and high hyperopia was defined as SE of +6.0 Diopters and more. Myopia progression was defined as the difference between the cycloplegic SE at the 5-year follow-up and at baseline.

Since there was a strong correlation between SE of the right and left eyes (r = 0.801, *p* < 0.0001), all data are presented for the right eye.

### 2.3. Measurement of Amplitude of Accommodation

All measurements of the amplitude of accommodation (AA) were taken before the instillation of eye drops and included push-up and push-down methods, which were performed with a 5 min interval to prevent visual fatigue. The Royal Air Force rule was utilized to measure the AA, and an overhead lamp was placed on the target’s top for adequate illumination. Under the push-up method, the target was held at a 40 cm distance and was moved closer to the eye until an individual reported blurring. Under the push-down method, the target moved away from the eye until an individual reported clarity. The target was moved with a constant speed equal to 5 cm per second. The tests were performed monocularly. Three measurements were made, and an average was calculated and recorded. The AA was considered to be the inverse of the distance from the target to the eye [15].

In addition to AA measurement, accommodative excess was determined by calculating the difference in SE between non-cycloplegic and cycloplegic autorefraction [16].

### 2.4. Evaluation of Risk Factors Associated with Myopia Progression

In consultation with international research [11,13,17,18], a self-administered questionnaire was constructed to evaluate the risk factors associated with myopia progression. To ensure the content validity of the questionnaire, three experts in the field—two consultant optometrists and one consultant ophthalmologist—reviewed the initial version. Their feedback ensured that the questionnaire comprehensively covered all relevant risk factors for myopia progression. Given the harsh climate conditions in East Kazakhstan, where people spend a significant amount of time indoors and there are few clear days throughout the year, it was decided to omit the question of the level of exposure to outdoor lighting. This decision was based on the rationale that the indoor lifestyle in this region would make such a question less relevant. To assess the reliability of the questionnaire, it was administered twice to a group of 26 fourth-year medical students at SMU, with a two-week interval between administrations. The consistency of responses over the two administrations was high, demonstrating good test–retest reliability. Cronbach’s alpha was calculated as a measure of internal consistency and equaled 0.713, indicating acceptable reliability.

The resulting questionnaire encompassed various risk factors, including age, gender, department at SMU, place of origin (urban v. rural), ethnicity, the number of children in the parental family, and birth order. It also inquired about the history of prematurity and birth traumas, discerning between their presence and absence. Furthermore, participants provided information on the myopic status of their parents, grandparents, siblings, aunts/uncles, and cousins. The questionnaire delved into subjective experiences of visual fatigue and ocular redness. Behavioral factors within the most recent year were also explored, such as self-reported time spent on activities such as reading, writing, handwork, watching television, computer use, mobile phone use, sleeping, and engagement in physical and outdoor activities.

### 2.5. Statistical Analysis

All statistical tests were conducted using the Statistical Package for Social Sciences (SPSS) software, version 20. Continuous variables were assessed for the normality of data distribution using the Lilliefors test and by computing histograms and Q-Q plots. Since the data exhibited a non-normal distribution, they were presented as median (Me) with 25th and 75th percentiles (Q1; Q3), and the Wilcoxon test was employed for between-group comparisons. Categorical variables were presented as absolute numbers and proportions (%). Multivariable logistic regression analysis was performed to assess the role of different risk factors for myopia progression at the 5-year follow-up. Two models were computed: under the first model, myopia progression ≥ 0.5 Diopters was considered, while under the second model, myopia progression ≥ 1.0 Diopters was considered. The variables included in the analysis comprised all questions from the self-administered questionnaire, as well as SE and accommodative excess in Diopters. Only the adjusted odds ratios (OR) with 95% confidence intervals (95% CI) are presented. The significance level for all statistical tests was set at <0.05.

## 3. Results

Table 1 presents the demographic characteristics of the study participants. The majority of students were female (61.8%) and belonged to the Kazakh ethnicity (61.8%). The most common education track was General Medicine (84.2%), followed by Dentistry (11.5%). Most students came from urban areas (70.7%) and were the firstborns in their families (54.9%).

In the first year of study, before cycloplegia, the median SE in the right eye was −0.75 Diopters, which decreased to −0.25 Diopters after cycloplegia. Out of 696 students, 440 (63.2%) had myopia before cycloplegia. This proportion decreased to 44.7% after cycloplegia, and the difference was significant. Similarly, the proportion of individuals with emmetropia decreased from 31.8% to 31.5% after cycloplegia. The proportion of individuals with hyperopia increased from 5% before cycloplegia to 23.8% after cycloplegia (Table 2).

When the students progressed to the fifth course, the median SE was −1.125 Diopters before cycloplegia, and it decreased to −0.5 Diopters after cycloplegia. As many as 70.4% of individuals had various degrees of myopia before cycloplegia, and this proportion decreased to 47.5% after cycloplegia. The proportion of individuals with emmetropia constituted 20.8% before cycloplegia and 30.3% after cycloplegia, and that of hyperopia constituted 2.8% and 16.2%, respectively (Table 3).

In the comparison of SE after cycloplegia between the same students in the first and fifth years, it became obvious that for low myopia, the change constituted −0.25 Diopters, which was significant, while for moderate myopia, the change constituted −0.5625 Diopters. The median SE for high myopia, emmetropia, and low hyperopia did not change when students progressed from the first to the fifth course (Table 4).

Table 5 presents comparisons of the AA and accommodative excess between first-year and fifth-year students. The difference between non-cycloplegic and cycloplegic refraction increased as students progressed from the first to the fifth course, and this change was significant. There was a significant difference in the AA measured by the push-down method and an insignificant difference in the AA measured by the push-up method, although the medians of the AA appeared to be the same.

Table 6 presents the results of a logistic regression analysis aimed at identifying factors associated with myopia progression at the 5-year follow-up. Two models were constructed: the first identified factors leading to myopia progression of ≥0.5 diopters, while the second aimed to identify factors leading to myopia progression of ≥1.0 diopters. Accommodative excess at the fifth course and spherical equivalent (SE) at the first course were significantly associated with myopia progression of both ≥0.5 and ≥1.0 diopters. Hours spent reading books, writing, working at a laptop/desktop, using a mobile device, and sleeping were positively and significantly associated with myopia progression of ≥0.5 diopters. For the progression of myopia to ≥1.0 diopters, age and hours spent on handwork were negative but insignificant influencing factors. Being from a rural area served as a significant protective factor, while having myopic cousins was an insignificant protective factor.

## 4. Discussion

This longitudinal study aimed to investigate the refractive status, AA, and myopia progression in Kazakhstani medical students over a 5-year follow-up period. To our knowledge, this is one of very few studies with such an extensive follow-up duration. In the first course, the median SE was −0.75 Diopters before cycloplegia and −0.25 Diopters after cycloplegia. In the fifth course, it constituted −1.125 Diopters before cycloplegia and −0.5 Diopters after cycloplegia. The proportion of students diagnosed with myopia following cycloplegic refraction increased from 44.7% in the first year to 47.5% in the fifth year. Conversely, the proportion of emmetropic students declined from 31.5% to 30.3%, and hyperopia decreased from 23.8% to 16.8% as students progressed from their first year of training to the fifth. Simultaneously, the dioptric power of accommodative excess increased from 0.375 in the first year to 0.50 in the fifth year. The hours spent on near-work activities, such as reading books, writing, working at a computer, and using a mobile device, were significantly associated with a myopia progression of ≥0.5 Diopters. The implications of these findings warrant a more comprehensive discussion.

Several international longitudinal studies with varying durations of follow-up have investigated the progression of myopia in medical students. Lv et al. conducted a study on Chinese medical students with a follow-up duration of 2 years. Similar to this study, all students underwent autorefraction in cycloplegia, and the prevalence of myopia at baseline was 78.5%, increasing to 84.1% at the 2-year follow-up. The mean refractive error at baseline was −2.52 Diopters, which increased to −2.84 Diopters over the 2-year period [10]. The observations made by Lv et al. align with other studies from China, where the prevalence of myopia is higher than in many other parts of the world. As a recent population-based study showed, as many as 75.35% of children and young adults were myopic [18]. However, the prevalence of myopia in Central Asia is one of the lowest in the world [1], which is also supported by our findings.

A longitudinal study on students of the School of Medicine in Indonesia with a 1-year follow-up revealed that at baseline, 69.4% of students had myopia, as determined with an autorefractor without cycloplegia. The average myopic shift per year was −0.401 Diopters, and the prevalence of myopia was significantly higher in students of Chinese origin compared to students of Javanese and other origins [19]. Although the rate of myopia reported by Nugroho et al. was higher than observed in our study, the comparability of the study by Nugroho et al. is limited to our findings since only non-cycloplegic refraction was measured.

In a 2-year longitudinal study on myopia progression in medical students conducted in Denmark, autorefraction in cycloplegia was employed. At baseline, the prevalence of myopia was 37%, and one year later, it increased to 43%. The mean SE was −0.50 at baseline and −0.74 at the 2-year follow-up [11]. Although the baseline rate of myopia was similar to our study, the rate of myopia progression was higher. This conclusion is supported by the fact that the study used a comparable methodology and definition of myopia (SE ≥ 0.5 Diopters).

In Turkey, cycloplegic autorefraction was utilized to determine the rate of myopia progression in medical students with a 1-year follow-up. The rate of myopia was 32.9%, with low myopia being the most common type (81%). However, the authors failed to establish myopia progression one year later, as the mean SE did not change significantly from baseline to one-year follow-up (SE = −0.67 Diopters v. SE = −0.65 Diopters) [20]. The failure to establish myopia progression at the 1-year follow-up aligns with our findings showing a consistent rate of myopia progression. Nevertheless, disparities in the study methodology exist, particularly in the use of a different definition of myopia (SE ≥ 0.75 Diopters).

Fesharaki et al. conducted a longitudinal study on the Iranian population of medical students with a comparable duration of follow-up, equal to 5.5 years. The prevalence of myopia at baseline was 46.5%, which increased to 64.0% during the study period, and the mean myopic shift constituted 0.20 Diopters per year [21]. It has to be noted that although the rate of myopia at baseline was comparable to our study, the authors used a different definition of myopia (SE ≥ 0.25 Diopters), and it is not clear whether refraction was measured with cycloplegia or not.

There is a scarcity of studies assessing AA in university students. Majumder and Afnan employed various methods to determine the AA in a small sample of students from a Malaysian Private University. The mean age of the participants was 22.26 years, and, as measured by the push-up method, the mean AA was 11.38 Diopters, while, as measured by the push-down method, it was 10.35 Diopters [22]. Mathebula et al., in a study on South African university students aged 21 to 27 years, reported a mean AA of 10.23 Diopters using the push-up method and 8.43 Diopters using the minus lens method [23]. The findings of our study align with previously published data, indicating that the AA measured by the push-up method results in a higher AA compared to the push-down method. It is widely acknowledged that the push-up method tends to overestimate AA, while the push-down method tends to underestimate it [16]. Consequently, these findings need to be interpreted with consideration of this known phenomenon.

In recent years, numerous studies have been conducted to assess the risk factors contributing to myopia progression, believed to result from a complex interplay of genetic, environmental, and behavioral factors. The role of genetic inheritance has been substantiated by both familial and genome-wide association studies, with behavioral factors such as prolonged near vision work, including excessive digital screen time, significantly contributing to myopia progression [24]. Notably, environmental factors such as increased time spent outdoors and exposure to light have been shown to mitigate the influence of parental myopia and near-work time [25]. Several studies investigated the risk factors for myopia progression in medical students. In a study by Jacobsen et al., time spent reading scientific literature and age were identified as risk factors for myopia progression, whereas physical activity was deemed a protective factor [11]. Conversely, the study by Onal et al. did not establish a significant difference in the amount of near-vision work between myopes and nonmyopes [20]. In our study, engaging in activities such as reading books, writing, and using a laptop/desktop and mobile devices showed a significant positive association with a myopia progression ≥ 0.5 Diopters. However, age demonstrated an insignificant association with a myopia progression ≥ 1.0 Diopters.

The findings of this study suggest implications for public health policy and educational practice. Given that increased near-work activity was significantly associated with myopia progression among medical students, there may be a need to reconsider the structure of medical education curricula. Integrating strategies to reduce the intensity and duration of visual workload, such as alternating periods of near vision work with tasks involving physical activity and promoting outdoor physical activity during study breaks, particularly in warmer seasons, could potentially mitigate the risk of myopia development and progression [26]. Furthermore, promoting vision preservation education and implementing regular vision screening protocols within medical schools could facilitate early detection of myopia among students. These proactive measures not only support the overall health and well-being of medical students but also underscore the importance of vision care as an integral component of medical education [27].

This study has several limitations, the most significant being its single-university focus. The involvement of other medical schools could potentially yield different findings. In addition, the questionnaire did not include an important risk factor: the level of exposure to outdoor light. The reason for this limitation is the limited amount of time that people living in this part of Kazakhstan spend outdoors due to a markedly continental climate with prolonged cold winters, brief summers, and a lack of clear days. Instead, this study emphasized the near-work activities, as the intensity of training in medical school is significant, and students dedicate a substantial amount of time to their studies, making increased near-work activity an important risk factor to investigate. Furthermore, the assessment of genetic factors relied solely on a self-administered questionnaire about spectacle wear, assuming that the students knew precisely why their relatives used spectacles. In reality, some might be aware of this, while others might not.

The study also did not find statistically significant effects for certain risk factors such as genetics and physical activity. This limitation may be partly attributed to the constraints of sample size. Future research would benefit from larger and more diverse samples involving several universities to address these limitations effectively. Utilizing advanced statistical techniques such as structural equation modeling or path analysis could allow for a more comprehensive exploration of the complex relationships among risk factors influencing myopia progression. Moreover, integrating objective measures alongside self-reported data could enhance the precision of assessing these risk factors.

However, the study also exhibits notable strengths, including a substantial sample size at both baseline and follow-up, as well as an extended duration of follow-up, rendering it unique among similar studies. Furthermore, the study stands out by measuring the AA at both baseline and follow-up, a feature uncommon in this type of research.

## 5. Conclusions

In comparison to other studies involving medical students, the baseline prevalence of myopia is relatively lower, and the observed myopic shift at the 5-year follow-up is less pronounced than anticipated based on existing research in this field. This disparity may be attributed to the influence of genetic factors, given the well-established lower prevalence of myopia in the Central Asian population. Alternatively, it is conceivable that the educational burden on medical students in Kazakhstan is comparatively lighter than that experienced by their counterparts in other regions globally.

The findings from this study validate several risk factors previously reported for myopia progression during young adulthood. However, certain established factors (such as hereditary influences and those related to physical activity) failed to demonstrate a significant impact, potentially due to the relatively modest sample size. Significantly, this study affirms that myopia can progress into the third decade of life, albeit with a generally smaller shift compared to the more substantial progression observed during childhood and adolescence. There is a pressing need for additional research into other factors contributing to myopia progression during adulthood, as well as the identification of young adults prone to rapid myopia progression.

## Figures and Tables

**Table 1:** Demographic characteristics of study participants (*n* = 696).

Variables		N	%
Age at first year of medical training, years (median; Q1; Q3)		18 (18; 19)	
Specialty	Medicine	586	84.2
	Public Health	16	2.3
	Dentistry	80	11.5
	Nursing	8	1.1
	Pharmacy	6	0.9
Sex	Male	266	38.2
	Female	430	61.8
Race (Ethnicity)	Asian (Kazakh)	571	82.0
	Caucasian (Russian, Ukrainian, German)	27	3.9
	South Asian (Indian)	90	12.9
	Other	8	1.1
Place of origin	Urban	492	70.7
	Rural	204	29.3
Number of children in a family	One-two	353	50.7
	Three-four	287	41.2
	Five-six	47	6.8
	Seven and more	9	1.3
Position in the birth order among siblings	First child	389	54.9
	Second-third child	282	40.5
	Fourth-fifth child	25	3.6
	Sixth child and more	7	0.9

**Table 2:** Spherical equivalent refraction of the right eye in the first-year students (*n* = 696).

Refractive Status		Before Cycloplegia N (%)	After Cycloplegia N (%)	*p*-Value **
SE *, Diopters (median; Q1; Q3)		−0.75 (−0.125; −1.875)	−0.25 (0.375; −1.5)	<0.001
Myopia	Low	334 (48.0)	218 (31.3)	<0.001
	Moderate	82 (11.8)	73 (10.5)	
	Severe	24 (3.4)	20 (2.9)	
Emmetropia		221 (31.8)	219 (31.5)	
Hyperopia	Low	34 (4.9)	162 (23.3)	
	Moderate	-	5 (0.4)	
	Severe	1 (0.1)	1 (0.1)	

*—Spherical equivalent. **—Wilcoxon test.

**Table 3:** Spherical equivalent refraction of the right eye in the fifth-year students (*n* = 655).

Refractive Status		Before Cycloplegia N (%)	After Cycloplegia N (%)	*p*-Value **
SE *, Diopters (median; Q1; Q3)		−1.125 (−0.375; −2.125)	−0.5 (0.25; −1.625)	<0.001
Myopia	Low	368 (52.9)	231 (33.2)	<0.001
	Moderate	92 (13.2)	77 (11.1)	
	Severe	30 (4.3)	22 (3.2)	
Emmetropia		145 (20.8)	211 (30.3)	
Hyperopia	Low	19 (2.7)	112 (16.1)	
	Moderate	1 (0.1)	1 (0.1)	
	Severe	-	1 (0.1)	

*—Spherical equivalent. **—Wilcoxon test.

**Table 4:** Comparison of spherical equivalent refraction of the right eye after cycloplegia between first-year and fifth-year students.

SE *, Diopters		First Year	Fifth Year	Change	*p*-Value **
Myopia, Median (Q1; Q3)	Low	−0.875 (−0.625; −1.625)	−1.125 (−0.750; −2.00)	−0.25	0.023
	Moderate	−4.3125 (−3.46875; −5.1875)	−4.875 (−3.34375; −5.75)	−0.5625	0.400
	High	−7.375 (−6.78125; −8.84375)	−7.375 (−6.8125; −8.6875)	0	-
Emmetropia, Median (Q1; Q3)		0.125 (−0.125; 0.250)	0.125 (−0.125; 0.250)	0	0.227
Hyperopia, Median (Q1; Q3)	Low	0.750 (0.50; 1.00)	0.750 (0.50; 1.00)	0	0.227
	Moderate	3.325 (3.125; 3.3125)	-	-	-
	High	-	-	-	-

*—Spherical equivalent. **—Wilcoxon test.

**Table 5:** Comparison of amplitude of accommodation and accommodative excess in the right eye between first-year and fifth-year students.

Accommodation, Diopters	First Year	Fifth Year	*p*-Value *
Accommodative excess Median (Q1; Q3)	−0.375 (−0.125; −0.75)	−0.50 (−0.125; −1.00)	<0.0001
Push-down Median (Q1; Q3)	3.00 (2.00; 3.00)	3.00 (2.00; 3.00)	0.032
Push-up Median (Q1; Q3)	4.00 (3.00; 6.00)	4.00 (3.00; 6.00)	0.129

*—Wilcoxon test.

**Table 6:** Logistic regression analysis to determine factors associated with myopia progression at 5-year follow-up.

Factors		Myopia Progression ≥ 0.5 Diopters			Myopia Progression ≥ 1.0 Diopters		
		β	OR (95% CI) *	*p*-Value	β	OR (95% CI)	*p*-Value
Age, years		-	-	-	−0.260	0.771 (0.585; 1.016)	0.065
Place of origin	Urban	Reference			Reference		
	Rural	-	-	-	−0.808	0.446 (0.235; 0.844)	0.013
Myopic cousin(s)	No	Reference			Reference		
	Yes	-	-	-	−0.779	0.459 (0.196; 1.0171)	0.072
Accommodative excess at the fifth course, Diopters		0.593	1.810 (1.259; 2.601)	0.001	1.484	4.411 (2.073; 9.382)	<0.0001
Handworks, hours per day		-	-	-	−1.059	0.347 (0.103; 1.168)	0.087
SE ** at the first course, Diopters		−0.276	0.759 (0.687; 0.838)	<0.0001	−0.327	0.721 (0.632;0.824)	<0.0001
Reading paper books, hours per day		0.211	1.234 (1.078; 1.414)	0.002	-	-	-
Writing, hours per day		0.170	1.186 (1.016; 1.384)	0.031	-	-	-
Laptop/desktop, hours per day		0.153	1.165 (1.017; 1.334)	0.027	-	-	-
Mobile, hours per day		0.163	1.177 (1.058; 1.309)	0.003	-	-	-
Sleep, hours per day		0.241	1.272 (1.065; 1.520)	0.008	-	-	-
Model parameters		−2 Log Likelihood = 605.025 Nagelkerke R^2^ = 0.173 *p* ≤ 0.0001			−2 Log Likelihood = 286.394 Nagelkerke R^2^ = 0.245 *p* ≤ 0.0001		

*—Odds Ratio with 95% Confidence Interval. **—Spherical Equivalent.

## Data Availability

The data presented in this study are available on request from the corresponding author. The data are not publicly available due to the protection of personal data.
